# Sleep Timing in Late Autumn and Late Spring Associates With Light Exposure Rather Than Sun Time in College Students

**DOI:** 10.3389/fnins.2019.00882

**Published:** 2019-08-28

**Authors:** Tamar Shochat, Nayantara Santhi, Paula Herer, Sapphira A. Flavell, Anne C. Skeldon, Derk-Jan Dijk

**Affiliations:** ^1^Cheryl Spencer Department of Nursing, Faculty of Social Welfare and Health Sciences, University of Haifa, Haifa, Israel; ^2^Surrey Sleep Research Centre, Faculty of Health and Medical Sciences, University of Surrey, Guildford, United Kingdom; ^3^Department of Mathematics, Faculty of Engineering and Physical Sciences, University of Surrey, Guildford, United Kingdom

**Keywords:** light, circadian, sleep, mathematical modeling, entrainment, chronotype

## Abstract

Timing of the human sleep-wake cycle is determined by social constraints, biological processes (sleep homeostasis and circadian rhythmicity) and environmental factors, particularly natural and electrical light exposure. To what extent seasonal changes in the light-dark cycle affect sleep timing and how this varies between weekdays and weekends has not been firmly established. We examined sleep and activity patterns during weekdays and weekends in late autumn (standard time, ST) and late spring (daylight saving time, DST), and expressed their timing in relation to three environmental reference points: clock-time, solar noon (SN) which occurs one clock hour later during DST than ST, and the midpoint of accumulated light exposure (50% LE). Observed sleep timing data were compared to simulated data from a mathematical model for the effects of light on the circadian and homeostatic regulation of sleep. A total of 715 days of sleep timing and light exposure were recorded in 19 undergraduates in a repeated-measures observational study. During each three-week assessment, light and activity were monitored, and self-reported bed and wake times were collected. Light exposure was higher in spring than in autumn. 50% LE did not vary across season, but occurred later on weekends compared to weekdays. Relative to clock-time, bedtime, wake-time, mid-sleep, and midpoint of activity were later on weekends but did not differ across seasons. Relative to SN, sleep and activity measures were earlier in spring than in autumn. Relative to 50% LE, only wake-time and mid-sleep were later on weekends, with no seasonal differences. Individual differences in mid-sleep did not correlate with SN but correlated with 50% LE. Individuals with different habitual bedtimes responded similarly to seasonal changes. Model simulations showed that light exposure patterns are sufficient to explain sleep timing in spring but less so in autumn. The findings indicate that during autumn and spring, the timing of sleep associates with actual light exposure rather than sun time as indexed by SN.

## Introduction

The light-dark cycle is considered the most important synchronizer of the human circadian pacemaker (Duffy and Wright, 2005) and together with sleep homeostasis, determines sleep propensity (Dijk and Czeisler, 1995). Sources of light available to humans are the natural light-dark cycle and “electrical” light. Even though the natural light-dark cycle is driven by geophysical cycles, exposure to natural light is largely determined by behavioral patterns, e.g., indoor work (Wright et al., 2013). Exposure to electrical light is also linked to behavioral cycles, the most prominent being the sleep-wake cycle, which is often dictated by social constraints such as work and school schedules (Wittmann et al., 2006). The interaction of light exposure, behavioral cycles, social constraints and sleep homeostasis has been summarized in mathematical models (Phillips et al., 2010; Skeldon et al., 2017).

Associations between habitual diurnal light exposure, sleep and circadian outcomes, in the “ecological” home environment, indicate that people with early timing of sleep and daytime activities (early types) have increased light exposure in the early morning hours, whereas late types have increased light exposure in the evening hours with a lower amplitude of the light-dark exposure cycle (Goulet et al., 2007; Emens et al., 2009; Martin et al., 2012; Van der Maren et al., 2018). The duration of bright light exposure (>1000 lux) is slightly higher in early compared to late types (Goulet et al., 2007; Emens et al., 2009), with a greater difference between the extreme morning and extreme evening types (Emens et al., 2009). A study of individuals with delayed sleep phase confirmed that relative to clock time, their light exposure patterns were shifted to later in the day compared to controls (intermediate sleep phase) but, relative to circadian time, i.e., dim light melatonin onset (DLMO), their light exposure occurred earlier than in controls (Wilson et al., 2018).

Young individuals commonly exhibit a delayed sleep phase (Hagenauer et al., 2009; Gradisar and Crowley, 2013) and consistently demonstrate large differences between sleep timing on weekdays and weekends (Roenneberg et al., 2004; Shochat et al., 2017). This is thought to reflect the biological shift toward later sleep timing that starts in early puberty and ends around age 20 (Roenneberg et al., 2004; Hagenauer et al., 2009). Developmental changes in the circadian clock, changes in sleep homeostasis, early school and work regimes, social forces including peer pressure, reduced daytime light and increased evening light exposure have all been implicated as causal factors (Crowley et al., 2018).

While many field studies examining associations between light, habitual sleep and circadian outcomes and individual differences therein, focus on the role of factors such as chronotype, they rarely consider the impact of weekday-weekend, seasonal changes, and changes between standard time (ST) and daylight saving time (DST) (Goulet et al., 2007; Emens et al., 2009; Burgess and Molina, 2014; Chinoy et al., 2018)^.^

Seasonal changes dictate differences in day length, which partly determines overall light exposure including its spectral composition (Thorne et al., 2009; Stothard et al., 2017) and thereby may affect sleep schedules. Later bed and wake times in winter relative to summer were observed in university students in Norway, where there are large seasonal differences in the daily photoperiod, but not in students from equatorial Ghana where there are no seasonal differences in photoperiod (Friborg et al., 2012). On the other hand, seasonal effects on sleep duration are very small, i.e., in the order of minutes (Allebrandt et al., 2014) or not statistically significant (Lo et al., 2014). In the latter paper, a comparison of weekday/weekend sleep schedules across seasons between participants from Singapore (very small changes in photoperiod, no DST) and Surrey (large changes in photoperiod and DST), indicated that both sleep timing and duration are primarily determined by social zeitgebers, e.g., clock time and associated light exposure rather than the natural light-dark cycle.

The impact of the timing of the natural light dark cycle relative to clock time has been derived from the study of spontaneous sleep timing (i.e., sleep timing during weekends) in individuals living at different longitudes within a time zone. People living on the west side of time zones where solar noon (SN) occurs at a later clock time, report sleeping later on free days compared to those living on the east side of the time zone (Roenneberg et al., 2007). These observations are in accordance with the notion that the human circadian system is entrained by “sun time” (Roenneberg et al., 2007).

In describing and interpreting changes in sleep timing in relation to light exposure and its changes across seasons, it is important to define the phase reference points used to quantify timing differences. When dawn or dusk are used as phase reference points, the timing of social constraints which for most people are phase-locked to clock time, change across seasons. Employing time elapsed from the middle of the dark period has been suggested to be a more informative reference point (Daan et al., 2002), or similarly, time elapsed from the middle of the light period, i.e., SN. SN, unlike dawn and dusk, does not depend strongly on photoperiod. SN remains at much the same clock time throughout the year in countries without DST. However, in countries with DST, the change from ST to DST is associated with an abrupt shift of 1 h for SN (and dawn and dusk) relative to clock time and associated social constraints. As an alternative to SN, dawn or dusk, the weighted midpoint of actual light exposure can be used as a phase reference point, or the entire time series of light can be entered into mathematical models aimed to assess the effects of light, using the model predicted minimum of the circadian oscillator as the reference point (Woelders et al., 2017). A prediction that follows from the notion that the human circadian timing system is entrained to sun time is that spontaneous sleep timing, as a proxy for circadian phase and quantified in local clock time, will be 1 h later during DST compared to ST. Alternatively, it may be argued that the human circadian timing system is entrained to social schedules and or actual light exposure, which is a combination of natural and electrical light, in which case spontaneous sleep timing will be associated with clock time or the timing of light exposure.

Ecological studies to date have yet to focus on the inter-individual variability of the timing of sleep and activity, and its association with light exposure on weekdays versus weekends and across seasons. Thus the aim of the present study in undergraduate university students in the United Kingdom was to assess sleep timing (i.e., bed, wake, and mid-sleep times), and timing of the midpoint of daily activity, on weekdays and weekends and in late autumn (ST) and in late spring (DST), relative to clock time, to SN, and to the timing of the weighted midpoint of light exposure, i.e., the time at which 50% of the total daily light exposure has accumulated (50%). Specifically we assessed (1) 24 h light exposure (LE), level and timing of 50% LE, by season (autumn/spring), day-type (weekday/weekend) and bedtime category (early/intermediate/late); (2) Bed, wake, mid-sleep and weighted midpoint (50%) of daily activity times by season, day-type and bedtime category, relative to clock time, SN, and 50% LE; and (3) we compared our experimental data to the predictions of the effects of light according to a mathematical model for the circadian and homeostatic regulation of sleep (Skeldon et al., 2017).

## Materials and Methods

### Ethics and Participants

The research protocol was approved by the University of Surrey Ethics Committee and was conducted in accordance with the principles of the Declaration of Helsinki. Written informed consent was obtained from all the participants prior to starting any study related procedures. Twenty-three healthy young men and women were recruited into the protocol after responding to local advertisements for participation in the study, and undergoing an initial phone screening. Inclusion criteria called for full-time university students residing in university dormitories or off- campus housing. Exclusion criteria (all ascertained with validated health and sleep questionnaires) included working night shifts, having or being currently treated for a sleep disorder or for depression, having ophthalmologic or other neurological abnormalities, acute or chronic illness, taking medications on a chronic basis, particularly medications affecting the central nervous system, alcohol intake >14 units per week on average and daily consumption of more than four cups of caffeinated beverages (e.g., coffee, tea, cola) over the preceding 1 month. Of the twenty-three participants, two did not complete the measurements and two were removed due to incomplete data, leaving 19 participants (mean age ± 1SD: 18.88 ± 0.83 years) who completed the study and were included in the analyses presented here.

### Study Design and Procedures

In this repeated measures observational study design, all measurements were collected during the late autumn of 2014 (November to December – starting at least 22 days after the change to ST, hereafter, autumn) and again in the late spring (April–May, 2015 – starting at least 29 days after the change to DST, hereafter, spring). Each assessment segment consisted of three-weeks (during the semester) of continuous monitoring of daily habitual light exposure and rest-activity patterns, self-assessed bed and wake times and evening sleepiness levels (these data are not discussed here). The 24-h light exposure and rest-activity cycles were recorded via actigraphy. Self-reported daily bedtimes and wake times were recorded with sleep diaries. Furthermore, seasonal differences in natural illuminance were recorded, with an actiwatch encased in a clear container, for two weeks during each assessment segment outdoors at 51°13′36″N 0°38′36″W, a green belt area without electrical light sources in its vicinity.

#### Assessments of Daily Light Exposure, Rest-Activity Patterns, and Sleep Patterns

##### Light exposure and activity

This was measured with Actigraphy technology (Actiwatch-L, CamNtech Ltd.). The Actiwatch-L is a wrist-worn ambulatory device designed for studies in naturalistic settings that features continuous objective recording of white light illuminance in units of lux, and activity data (wrist movements at minute resolution) that is scored as epochs of sleep and wake, concomitantly. We computed daily light exposure and activity levels described below from these data.

##### Sleep

The Karolinska Sleep Diary was used for assessing daily sleep patterns. This self-report sleep diary provides information on self-selected daily bed and wake-times.

#### Deriving Light, Activity and Sleep Measures

##### Light

###### Daily (24-h) light exposure (24-h LE)

These are the daily (midnight to midnight) hourly averages computed individually for every participant. The data were log-transformed for analyses because the circadian effectiveness of light is linearly related to the log of light intensity over a wide range of intensities (Zeitzer et al., 2000).

###### Solar noon (SN)

The clock time at which the sun crossed the local meridian and reached the highest point. SN data was collected for all participants per study day from: https://www.timeanddate.com/sun/uk/guildford.

###### Midpoint of light exposure (50% LE)

This is the time at which 50% of the daily light exposure had accumulated. This was computed as half the cumulative light exposure (using log-transformed light levels) between wake time and bedtime, and was done for each study day for every participant.

##### Activity

###### Daily (24-h) activity levels

These are the daily (midnight to midnight) hourly averages computed individually for every participant.

###### Midpoint of activity (50% act)

This is the time at which 50% of the daily cumulative activity was reached. 50% of the daily activity level was computed as half the cumulative activity level between wake time and bedtime, and was done for each study day individually for every participant.

##### Sleep

###### Bedtime (BT)

These are self-reported bedtimes recorded in daily sleep diaries. Those from Sunday through Thursday were designated as weekday times, while those from Friday through Saturday were designated as weekend times.

###### Wake time (WT)

These are self-reported wake times recorded in daily sleep diaries. Those from Monday through Friday were designated as weekday times, while those from Saturday through Sunday were designated as weekend times.

###### Sleep duration (SD)

This was computed as the time elapsed between BT and WT.

###### Mid-sleep (MS)

This midpoint of the time between sleep onset (sleep diary) and wake time (WT) (sleep diary), and was computed for each study day individually for every subject. Mid-sleep (MS) has been proposed to be a proxy for circadian phase (Kantermann and Burgess, 2017).

###### BT category (BTcat)

Participants were divided to BT categories based on their weekday bedtimes during the autumn using the following cutoffs: Early (E):<=23:30 (*n* = 8); Intermediate (I): between 23:30 and 01:00 (*n* = 4), and Late: (L):>=01:00 (*n* = 7).

The sleep measures were aligned to local clock time, to SN and to 50% LE for the analyses.

#### Statistical Analyses

All the data were analyzed using the statistical package SAS 9.4 (SAS Institute Inc., Cary, NC, United States). For daily light and activity levels and for BT, WT, MS and 50% act (aligned to local clock time, SN and 50% LE) we used general linear mixed model (Procedure MIXED, SAS 9.4) ANOVA, with two levels each for season (spring and autumn) and day-type (weekday and weekend). The ML estimation method was used with a compound symmetry covariance structure for the subject effect. For all of the above variables, we then added 3 levels for the between subjects factor BTcat (described above) to the mixed model. The ML estimation method was used with compound symmetry covariance structure for the subject effect and unstructured covariance for the within-subjects effect. To ascertain individual variability we computed Intra Class Correlations (ICC) using the covariance parameter estimates from the mixed model ANOVA. ICC = CS (compound symmetry estimate) subject/(CS subject + residual). The significance of associations between the various measures were ascertained with Pearson’s correlations computed using Procedure Corr (SAS 9.4; SAS Institute Inc., Cary, NC, United States) and testing the null hypothesis of significance.

#### Model Simulations

Light profiles during autumn and spring and weekends and weekdays, averaged across participants, were used as input to the mathematical model reported in Skeldon et al. (2017). The model includes the entrainment of the circadian pacemaker by light and the homeostatic and circadian regulation of sleep. This model also describes the neuronal flip-flop switch that results in consolidated sleep and wake states. Switching between states is driven by circadian rhythmicity and sleep homeostasis. The mathematical model is an adaptation of the model of Phillips et al. (2010), incorporating social constraints and calibrated to make quantitative predictions of sleep-wake timing given realistic light profiles.

##### Physiological parameters for model simulations

The model includes three physiological parameters that are particularly relevant for determining individual differences in sleep duration and timing. These are: intrinsic circadian period, the rate of homeostatic rise during wake and circadian amplitude. As default values, we take the intrinsic circadian period as 24.2 h, consistent with the measured mean value (Duffy et al., 2011). We have selected the rate of homeostatic rise during wake and circadian amplitude to give a spontaneous sleep duration of 8.9 h and mid-sleep time of 05:00 for an intrinsic circadian period of 24.2 h and the average light profile used in Skeldon et al. (2017). These match the average sleep duration and timing for age 19 years reported in Roenneberg et al. (2004) and Roenneberg (2013). The rationale for fitting age based on homeostatic rise during wake and circadian amplitude is discussed in Skeldon et al. (2016).

##### Social constraints

Sleep timing is determined by the interplay of social and physiological factors. Given the differences between measured wake and bed times between weekdays and weekends, we make the assumption that wake during the week is determined by social factors and require that the model wake time occurs no later than the measured wake time. We then predict wake times at the weekend and sleep time, circadian phase and sleepiness for all days.

## Results

### Overall Effects of Day-Type and Season

**FIGURE 1:**
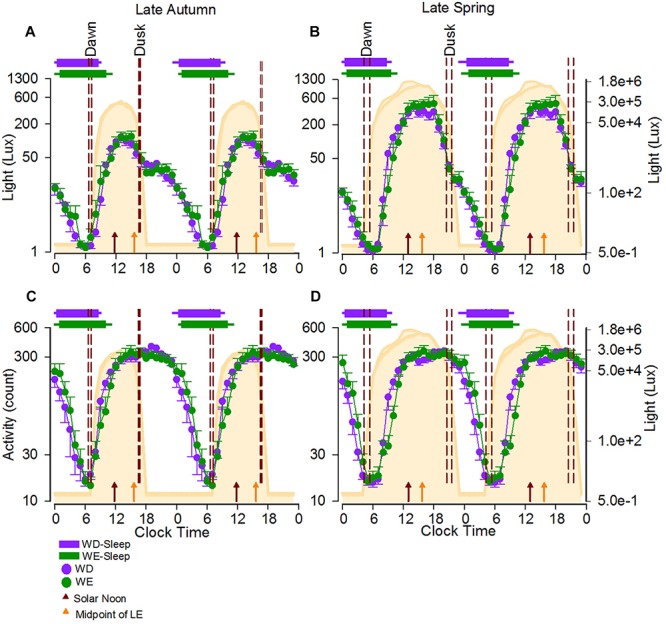
24-h Activity and light exposure profiles during weekdays and weekend in the late autumn and late spring. The average 24-h light **(A,B)** and activity **(C,D)** profiles of participants during the two seasons are shown as double plots in the figure. The area plot in the background of all four panels represents an average 24-h profile of natural illuminance during the two seasons. The horizontal bars show the average weekday (purple) and weekend (green) sleep, respectively, with the left and right error bars indicating the standard error of bed and wake times, respectively. The dashed reference lines indicate the dawn and dusk ranges during the study period. The brown and orange arrows represent the average SN and the average midpoint of light exposure times. The horizontal-axis represents clock time and the left vertical -axis represents light counts **(A,B)** and activity levels **(C,D)**. In both panels the right vertical axis represents light levels corresponding to the natural illuminance.

The natural light-dark cycle and average light exposure, activity and sleep timing during weekdays and weekends during autumn and spring are displayed in Figure 1.

#### Light Exposure (24-h and 50% LE) and Activity Levels (24-h)

**FIGURE 2:**
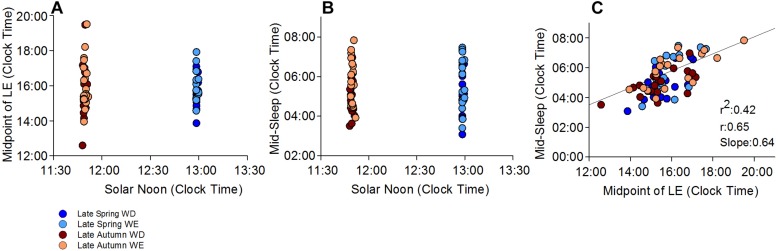
Midpoint of light exposure (50% LE) and mid-sleep time relative to SN and clock time. **(A)** shows individual 50% LE times relative to SN in late autumn and late spring. **(B)** shows individual mid-sleep times during weekends, weekdays in both seasons relative to the SN. **(C)** shows the relationship between 50% LE time and mid-sleep time, relative to clock time on averaged weekdays and weekends and in both seasons. Mid-sleep time is significantly correlated with the 50% LE exposure time, which explains 42% of the variance in mid-sleep time

Participants were exposed to light for a period much longer than the natural photoperiod on both weekdays and weekends, in both autumn and spring (Figures 1A,B). While the daily (24-h) light exposure level did not differ between day-types, it was higher in spring than in autumn (Table 1, and Figures 1A,B). SN was obviously later in spring (DST) (12:58, range 12:58–13:01) than in autumn (ST) (11:50, range 11:46–11:55). The midpoint of light exposure (50% LE) ranged from early afternoon to evening in both seasons (Figure 2A). Relative to clock time, 50% LE occurred after SN (Figure 1) and significantly later on weekends compared to weekdays (*p* = 0.01), with no seasonal differences (Table 1). Relative to SN, 50% LE occurred earlier in spring than in autumn on both weekdays and weekends (Table 1). Like light exposure, participants’ activity period was longer than the natural photoperiod on weekdays and weekends, particularly in autumn (Figures 1C,D). The average daily activity level (24-h) was higher on weekdays compared to weekends in both seasons (Table 1).

**TABLE 1:** Averages, standard errors of the mean (SEs) and intra-class correlation coefficients (ICCs) of 24-h log lux (and *retransformed*), 24-h activity counts, and timing of 50% of individual daily light exposure (50% LE).

					**DAY-TYPE**					**SEASON**			**DAY- TYPE^∗^**
	**Weekday**		**Weekend**		**difference**	**Late Autumn**		**Late Spring**		**Difference**	**DAY-TYPE**	**SEASON**	**SEASON**
					**Mean ± SE**					**Mean ± SE**			
	**LSmean ± SD**	**ICC**^#^	**LSmean ± SD**	**ICC**^#^	**(*n* = 19)**	**LSmean ± SD**	**ICC**^#^	**LSmean ± SD**	**ICC**^#^	**(*n* = 18)**			
24-h Light	0.99 ± 0.15	0.02	0.93 ± 0.20	<0.01	0.06 ± 0.04	0.85 ± 0.18	0.02	1.07 ± 0.17	0.02	-0.22 ± 0.04	*F*(1,70) = 1.79	*F*(1,70) = 24.15	*F*(1,70) = 0.03
(log of lux and	*9.77*		*8.51*			*7.08*		*11.75*					
*retransformed*)						WD: 0.87 ± 0.18		WD:1.10 ± 0.18^∗^			*p* = 0.1852	*p* < 0. 0001	*p* = 0.8616
						*7.41*		*12.59*					
						WE: 0.82 ± 0.26		WE:1.03 ± 0.25^∗^					
						*6.61*		*10.72*					

24-h Activity	2.04 ± 0.13	0.03	1.99 ± 0.14	0.03	0.04 ± 0.01	2.02 ± 0.13	0.02	2.02 ± 0.13	0.03	−0.00 ± 0.02	*F*(1,70) = 6.24	*F*(1,70) = 0.00	*F*(1,70) = 0.27
(counts)						WD: 2.04 ± 0.14		WD: 2.03 ± 0.14			*p* = 0.0148	*p* = 0.9834	*p* = 0.6023
						WE: 1.99 ± 0.15^∗∗^		WE: 2.00 ± 0.14					

50% LE	15:32 ± 0:48	0.15	15:57 ± 0:54	0.15	−00:26 ± 0:09	15:45 ± 0:51	0.11	15:44 ± 0:50	0.23	00:04 ± 0:10	*F*(1,70) = 6.83	*F*(1,70) = 0.00	*F*(1,70) = 0.47
(hh:mm)						WD: 15:29 ± 0:54		WD:15:35 ± 0:53			*p* = 0.0110	*p* = 0.9897	*p* = 0.4948
						WE:16:00 ± 1:05^∗∗^		WE:15:54 ± 1:02					

50% LE	3.14 ± 0.78	0.12	3.55 ± 0.88	0.12	−0.36 ± 0.15	3.93 ± 0.83	0.11	2.76 ± 0.81	0.22	1.11 ± 0.16	*F*(1,70) = 6.84	*F*(1,70) = 53.70	*F*(1,70) = 0.40
(hrs after SN)						WD: 3.67 ± 0.87		WD:2.61 ± 0.86^∗^			*p* = 0.0109	*p* < 0.0001	*p* = 0.5312
						WE: 4.18 ± 1.06^∗∗^		WE:2.92 ± 1.00^∗^					

*^#^Intraclass correlation (ICC) for sleep diary data was computed via SAS proc mixed using the maximum likelihood method. ^∗^Differences between autumn and spring ^∗∗^Weekday (WD) - Weekend (WE) differences within season.*

#### Timing of Sleep and Activity Relative to Clock Time, SN and 50% LE

The timing of sleep and activity occurred later on weekends than on weekdays, but with some seasonal differences.

*Relative to clock time*, there was a main effect of day-type (*p* < 0.01) on sleep and activity times, with BT, WT, MS, and 50% act occurring later on weekends compared to weekdays (Table 2 and Figure 1). There were no seasonal effects on these measures, with the exception of WT which occurred significantly later on weekends in autumn compared to weekends in spring (day-type by season interaction: *p* = 0.03). There was a main effect of day-type (*p* < 0.01) on SD in that it was longer on weekends in both seasons (Table 2).

**TABLE 2:** Averages, standard errors of the mean (SEs) and intra-class correlation coefficients (ICCs) of sleep and activity measures by Clocktime.

					**Day-type**					**Season**			**Day-type^∗^**
	**Weekday**		**Weekend**		**difference**	**Late Autumn**		**Late Spring**		**difference**	**Day-type**	**Season**	**Season**
					**Mean ± SE**					**Mean ± SE**	***F*(df1, df2)**	***F*(df1, df2)**	***F*(df1, df2)**
	**LSmean ± SD**	**ICC^#^**	**LSmean ± SD**	**ICC**	**(*n* = 19)**	**LSmean ± SD**	**ICC**	**LSmean ± SD**	**ICC**	**(*n* = 18)**	***P*-value**	***P*-value**	***P*-value**
BT	00:33 ± 1:14	0.46	01:07 ± 1:16	0.40	−00:34 ± 00:09	00:52 ± 1:15	0.27	00:49 ± 1:13	0.61	00:01 ± 0:10	*F*(1,70) = 15.83	*F*(1,70) = 0.08	*F*(1,70) = 0.35
(hh:mm)						WD: 00:32 ± 1:17		WD: 00:34 ± 0:18			*p* = 0.0002	*p* = 0.7726	*p* = 0.5579
						WE: 01:11 ± 1:21^∗∗^		WE: 01:04 ± 0:19^∗∗^					

WT	8:49 ± 0:52	0.24	9:53 ± 0:55	0.32	−01:04 ± 00:09	9:26 ± 0:53	0.17	9:16 ± 0:52	0.31	00:02 ± 0:08	*F*(1,70) = 57.77	*F*(1,70) = 1.34	*F*(1,70) = 4.79
(hh:mm)						WD: 8:44 ± 0:56		WD: 8:53 ± 0:56			*p* < 0.0001	*p* = 0.2504	*p* = 0.0320
						WE: 10:07 ± 1:01^∗∗^		WE: 9:38 ± 1:00^*,^^∗∗^					

Mid-sleep (MS)	4:58 ± 0:57	0.45	5:46 ± 0:59	0.47	−00:48 ± 0:07	5:23 ± 0:57	0.35	5:21 ± 0:55	0.51	00:02 ± 0:08^1^	*F*(1,68) = 47.69	*F*(1,68) = 0.09	*F*(1,68) = 1.69
(hh:mm)						WD: 4:55 ± 1:00		WD:5:02 ± 0:57			*p* < 0.0001	*p* = 0.7626	*p* = 0.1976
						WE:5:52 ± 1:03^∗∗^		WE:5:40 ± 1:00^∗∗^					

50% act	16:23 ± 1:01	0.24	16:57 ± 1:09	0.10	−00:38 ± 0:13	16:49 ± 0:55	0.19	16:31 ± 1:01	0.19	00:17 ± 00:13^2^	*F*(1,54) = 7.29,	*F*(1,54) = 1.63,	*F*(1,54) = 0.04,
(hh:mm)						WD: 16:33 ± 0:57		WD: 16:13 ± 0:14			*p* = 0.0093	*p* = 0.2075	*p* = 0.8408
						WE: 17:04 ± 1:11		WE:16:49 ± 0:17^∗∗^					

Sleep	455.3 ± 65.8	0.33	489.0 ± 69.4	0.20	−32.9 ± 11.9	476.7 ± 67.2	0.32	467.7 ± 64.7	0.18	4.4 ± 7.9^1^	*F*(1,68) = 11.86	*F*(1,68) = 0.82	*F*(1,68) = 1.36
Duration						WD:454.2 ± 70.2		WD: 456.6 ± 67.7			*p* = 0.0010	*p* = 0.3698	*p* = 0.2472
(SD) (min)						WE:499.2 ± 76.4^∗∗^		WE: 478.8 ± 74.0					

*^#^Intraclass correlation (ICC) for sleep diary data was computed via SAS proc mixed using the Maximum likelihood method. ^∗^Differences between autumn and spring, ^∗∗^Weekday (WD) - Weekend (WE) differences within season. ^1^*n* = 17 ^2^*n* = 11.*

*Relative to SN* (Table 3), there were main effects of day-type (*p* < 0.01) and season (*p* < 0.0001) on BT, WT, MS, and 50% act, in that they occurred significantly later on weekends than on weekdays and earlier in spring (DST) than in autumn (ST). For WT there was a day-type by season interaction, so that weekday-weekend differences were larger in autumn than in spring.

**TABLE 3:** Averages and standard errors of the mean (SEs) of sleep timing (BT, WT, MS) and 50% activity after SN.

			**Day-type**			**Season**			**Day-type^∗^**
	**Weekday**	**Weekend**	**difference**	**Late Autumn**	**Late Spring**	**difference**	**Day-type**	**Season**	**Season**
			**Mean ± SE**			**Mean ± SE**	***F*(df1, df2)**	***F*(df1, df2)**	***F*(df1, df2)**
	**LSmean ± SD**	**LSmean ± SD**	**(*n* = 19)**	**LSmean ± SD**	**LSmean ± SD**	**(*n* = 18)**	***P*-value**	***P*-value**	***P*-value**
BT (hours	12.15 ± 1.23	12.72 ± 1.27	−0.57 ± 0.15	13.04 ± 1.25	11.84 ± 1.22	1.16 ± 0.17	*F*(1,70) = 15.77,	*F*(1,70) = 69.16,	*F*(1,70) = 0.32,
after SN)				WD: 12.71 ± 1.29	WD: 11.60 ± 1.26^∗^		*p* = 0.0002	*p* < 0.0001	*p* = 0.5717
				WE: 13.36 ± 1.35^∗∗^	WE: 12.08 ± 1.33^∗^,^∗∗^				

WT	20.41 ± 0.86	21.48 ± 0.81	−1.07 ± 0.14	21.60 ± 0.88	20.28 ± 0.87	1.18 ± 0.14	*F*(1,70) = 57.40,	*F*(1,70) = 85.88,	*F*(1,70) = 4.68,
(hours				WD: 20.91 ± 0.94	WD: 19.90 ± 0.93^∗^		*p* < 0.0001	*p* < 0.0001	*p* = 0.0339
after SN)				WE: 22.29 ± 1.02^∗∗^	WE: 20.67 ± 1.01^*,^^∗∗^				

Mid-sleep	16.70 ± 0.92	17.04 ± 0.95	−0.33 ± 0.11	17.41 ± 0.93	16.33 ± 0.89	1.13 ± 0.14^1^	*F*(1,68) = 9.72,	*F*(1,68) = 92.07,	*F*(1,68) = 1.35,
(MS)				WD:17.30 ± 0.96	WD:16.09 ± 0.92^∗^		*p* = 0.0027	*p* < 0.0001	*p* = 0.2491
(hours after SN)				WE: 17.52 ± 1.03	WE:16.56 ± 0.98^*,^^∗∗^				

50% act	3.95 ± 0.99	4.53 ± 1.13	−1.48 ± 0.20	4.97 ± 0.88	3.50 ± 0.99	0.57 ± 0.21^2^	*F*(1,54) = 8.26,	*F*(1,54) = 44.48,	*F*(1,54) = 0.08,
after SN				WD:4.71 ± 0.91	WD:3.18 ± 1.04^∗^		*p* = 0.0058	*p* < 0.0001	*P* = 0.7840
(hrs)				WE:5.24 ± 1.15	WE:3.82 ± 1.20^*,^^∗∗^				

*^∗^Differences between autumn and spring. ^∗∗^Weekday (WD) - Weekend (WE) differences within season. ^1^*n* = 17 ^2^*n* = 11.*

*Relative to 50% LE* (Table 4), there was a significant effect of day-type (*p* < 0.05) only on WT and MS, both of which were significantly later on weekends than on weekdays. MS, in both seasons, was distributed between 03:00–08:00 (Figure 2B). A significant correlation between MS and 50% LE explained 42% of the variance in MS, in that a later MS was associated with a later 50% LE (Figure 2C).

**TABLE 4:** Averages and standard errors of the mean (SEs) of sleep timing (BT, WT, MS) and 50% activity after 50% LIGHT EXPOSURE (50% LE).

			**Day-type**			**Season**			**DAY-TYPE^∗^**
	**Weekday**	**Weekend**	**difference**	**Late Autumn**	**Late Spring**	**difference**	**DAY-TYPE**	**SEASON**	**SEASON**
			**Mean ± SE**			**Mean ± SE**	***F*(df1, df2)**	***F*(df1, df2)**	***F*(df1, df2)**
	**LSmean ± SD**	**LSmean ± SD**	**(*n* = 19)**	**LSmean ± SD**	**LSmean ± SD**	**(*n* = 18)**	***P*-value**	***P*-value**	***P*-value**
BT after 50%	9.08 ± 1.01	9.21 ± 1.11	−0.12 ± 0.18	9.18 ± 1.06	9.11 ± 1.03	0.08 ± 0.16	*F*(1,70) = 0.57,	*F*(1,70) = 0.20,	*F*(1,70) = 0.05,
LE (hrs)				WD: 9.14 ± 1.09	WD: 9.02 ± 1.07		*p* = 0.4541	*p* = 0.6567	*p* = 0.8158
				WE: 9.23 ± 1.28	WE: 9.19 ± 1.22				

WT after 50%	17.28 ± 0.83	17.86 ± 0.95	−0.59 ± 0.17	17.61 ± 0.90	17.53 ± 0.87	0.02 ± 0.23	*F*(1,70) = 8.64,	*F*(1,70) = 0.17,	*F*(1,70) = 0.79,
LE (hrs)				WD: 17.23 ± 0.97	WD: 17.33 ± 0.95		*p* = 0.0045	*p* = 0.6805	*p* = 0.3767
				WE: 17.99 ± 1.20^∗∗^	WE: 17.73 ± 1.13				

Mid-sleep(MS)	13.44 ± 0.71	13.80 ± 0.86	−0.37 ± 0.16	13.66 ± 0.79	13.59 ± 0.75	0.04 ± 0.18^1^	*F*(1,68) = 4.49,	*F*(1,68) = 0.16,	*F*(1,68) = 0.01,
after 50%				WD:13.47 ± 0.81	WD:13.41 ± 0.79		*p* = 0.0377	*p* = 0.6872	*p* = 0.9351
LE (hrs)				WE: 13.85 ± 1.07	WE:13.76 ± 1.00				

50% act after	0.93 ± 0.69	0.97 ± 0.88	−0.02 ± 0.18	1.09 ± 0.68	0.81 ± 0.71	0.43 ± 0.18^2^	*F*(1,54) = 0.06,	*F*(1,54) = 2.05,	*F*(1,54) = 3.02,
50% LE				WD:1.23 ± 0.68	WD:0.67 ± 0.74^∗^		*p* = 0.8143	*p* = 0.1583	*p* = 0.0878
				WE:0.95 ± 0.98	WE:0.99 ± 0.95				

*^∗^Differences between autumn and spring. ^∗∗^Weekday (WD) − Weekend (WE) differences within season. ^1^*n* = 17 ^2^*n* = 11.*

### Individual Differences in Sleep Timing: Stability Across Seasons and Day-Type

**FIGURE 3:**
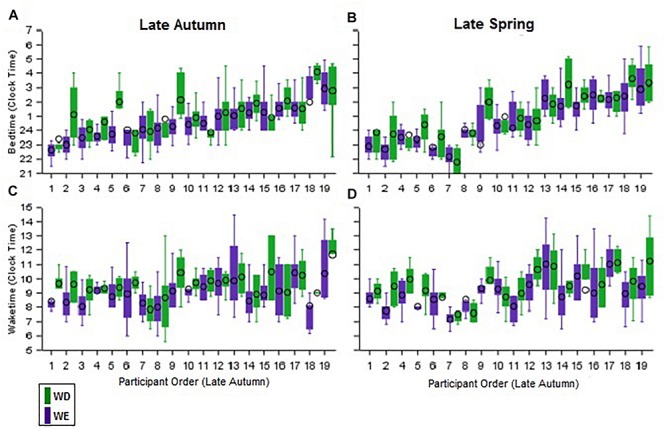
Inter individual differences and intra-individual stability in bed and wake times during weekdays and weekend in the late autumn and late spring. The daily bedtimes of participants during both seasons are shown as boxplots in the two upper panels **(A,B)** while the corresponding wake-times are shown in the two lower panels **(C,D)**. The purple boxplots represent weekdays and the green boxplots the weekend days. Participants in all four panels are ordered from earliest to latest weekday autumn bedtimes.

Individual differences in sleep timing (BT and WT) are illustrated in Figure 3. In all four panels, participants are ordered according to bedtimes on weekdays in autumn. The top panels show the within-individual consistency of BT across day-type and season, while the bottom panels show this in WT. The order of BT (earliest to latest, across participants) is not maintained in WT, indicating that BT is not a good predictor of WT. This was confirmed by a correlation analyses, in which BT-WT correlations by season and day-type did not reach significance (Supplementary Table S3). However, highly significant correlations for mean and median BT, WT, and MS between weekday and weekend by season (Supplementary Table S1), and between autumn and spring by day-type (Supplementary Table S2) and the intraclass correlations (Table 2) indicate that the BT and WT patterns within individuals are consistent across day-type and season.

**FIGURE 4:**
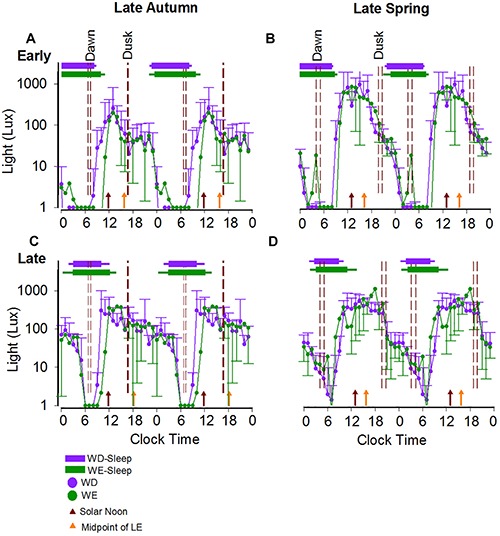
Weekday/weekend 24-h light exposure profiles and sleep timing during late autumn and late spring in a representative early and a representative late bedtime participant. The average 24-h light profiles of a participant with early **(A,B)** and late **(C,D)** sleep timing during both seasons are shown as double plots in the figure. The horizontal bars show the average weekday (purple) and weekend (green) sleep, respectively, with the left and right error bars indicating the standard deviations of bed and wake times, respectively. The dashed reference lines indicate the dawn and dusk ranges during the study period. The brown and orange arrows represent the average SN and the average midpoint of light exposure times. The horizontal-axis represents clock time.

Figure 4 shows light levels in two individual participants, one with early BT (A,B) and one with late BT (C,D) on weekdays and weekends in autumn (A,C) and in spring (B,D). Photoperiods clearly occur at a later clock time in the late BT participant than in the early BT participant.

Given this individuality, we analyzed light, sleep and activity timing measures across three bedtime categories (BTcat: early, intermediate and late sleepers) based on their weekday bedtimes in autumn (see cutoffs in section “Materials and Methods”). These measures were all analyzed relative to clock time, SN and 50% LE (Supplementary Tables S4–S6).

### Light, Sleep and Activity in the Three Bedtime Categories (BTcat)

#### Daily Light Exposure (24-h and 50%) and Activity (24-h)

**FIGURE 5:**
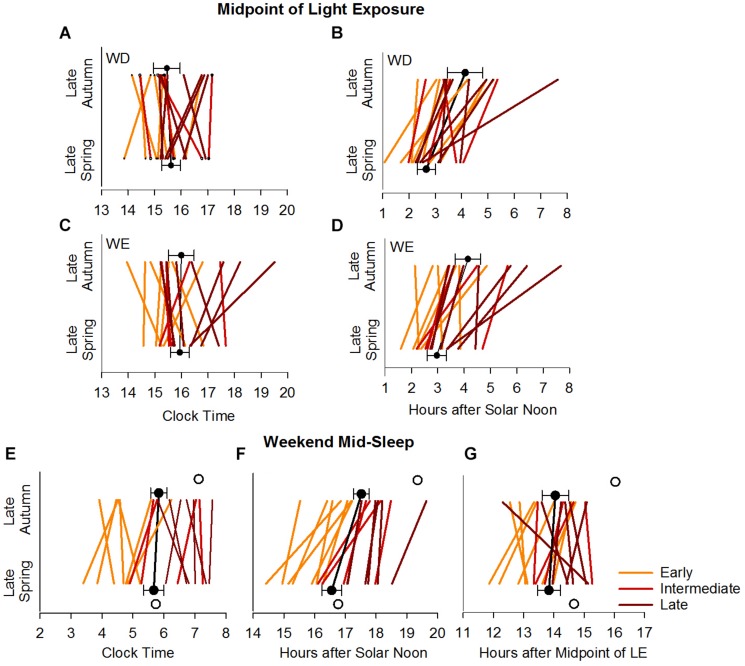
Midpoint of light exposure (50% LE) and weekend mid-sleep time in the late autumn and late spring. Upper panels show the individual and average weekday **(A,B)** and weekend **(C,D)** 50% LE times relative to clock time **(A,C)** and SN **(B,D)**. Lower panels show the individual and average weekend mid-sleep times during the late autumn and late spring relative to clock time **(E)**, solar noon **(F)** and midpoint of light exposure **(G)**. In all the panels, individuals are color coded for early, intermediate and late bedtime categories. The filled symbols with error bars represent the average times with between subjects variance (SEM). The open symbols (lower panels) represent the model predicted average mid-sleep times.

There was a significant main effect of BTcat (*F*_(__2__,__64__)_ = 6.94, *p* = 0.0019) on the daily 24-h LE exposure (in log lux), in that light levels (mean ± SE) were higher in the early: (1.02 ± 0.04) and late (1.00 ± 0.04) compared to the intermediate (0.77 ± 0.06) types. Figure 5A summarizes the seasonal and day-type differences in 50% LE, in the three bedtime categories, by clock time and by hours after SN. There were significant main effects of Btcat on the 50% LE, in that it occurred at a later clock time (*F*_(__2__,__64__)_ = 4.87, *p* = 0.0108) and at a later time compared to SN (*F*_(__2__,__64__)_ = 4.57, *p* = 0.0140) in the late types (mean ± SE for clock time: 16:14 ± 0:14 and for hours after SN: 3.81 ± 0.24) than in the early types (mean ± SE for clock time: 15:22 ± 0:14 and for hours after SN: 2.84 ± 0.23). Note that consistent with Figure 5A, relative to SN, the 50% LE occurred earlier in spring than in autumn both on weekdays and weekends, but relative to clock time it does not. For 24-h activity, no main effects and no interactions were found. 24-h light exposure and activity profiles in the three bedtime categories are shown in Supplementary Figures S1, S2, respectively.

#### Timing of Sleep and Activity Relative to Clock Time, SN and 50% LE

The results of the analyses of sleep timing measures (BT, WT, MS) and 50% act by BTcat relative to clock time, SN and 50% LE are shown in Supplementary Tables S4–S6, respectively. Overall, main effects for BTcat were observed for the timing of sleep and activity (with the exception of WT and 50% act relative to 50% LE) such that timing measures occurred at a later time from the early to the late BTcat (see pairwise comparisons for BTcat beyond season and day-type, and BTcat within season in the Tables). The main effects of season and day-type were in accordance with the pattern observed in Tables 2–4 (in general, later times on weekends compared to weekdays and earlier in the spring than in autumn).

Figure 5B summarizes seasonal differences in MS during the weekend, for the three bedtime categories, by clock time, hours after SN, and hours after 50% LE. Consistent with the above results, relative to SN the weekend MS occurred earlier in spring than in autumn, but relative to clock time and 50% LE, MS was unchanged across seasons.

### Simulating the Effects of Light Exposure on the Timing of Sleep the Timing of the Maximum of Circadian Sleep Propensity

**FIGURE 6:**
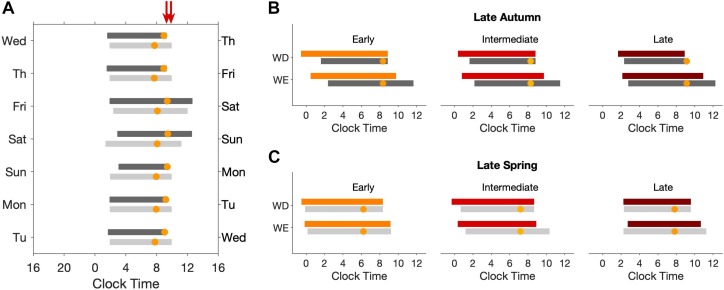
Model predicted weekday and weekend sleep times for early, intermediate and late types in the late autumn and late spring. Raster plots **(A)** for sleep timing in late autumn (dark gray bars) and late spring (light gray bars) for intermediate types. The left/right hand colored arrows indicate weekday wake time during spring/autumn. **(B,C)** show mean computed sleep timing for weekday and weekend for early, intermediate and late groups during the late autumn (dark gray bars) and late spring (light gray bars). For comparison, the measured values are also shown (colored bars). For all panels, the time of the computed circadian minimum wake propensity is indicated by the orange circles.

We next explored how current models of entrainment of the human circadian timing system respond to the observed light exposure patterns in the three BTcat. Figure 6A shows predicted sleep timing, and the timing of the minimum of circadian wake propensity, across the week for the light exposure patterns measured in spring and autumn for the intermediate bedtime category. The combination of the social constraints on wake time during the week and light exposure results in entrainment to a weekly pattern, with bedtime shifting slightly earlier Monday to Thursday as a consequence of increasing sleep debt. On Friday, in spite of a continued rise in sleep debt, bedtime is later than on Thursday. This is the result of entrainment to a weekly schedule leading to a small amplitude weekly oscillation in circadian timing with the circadian minimum of the wake propensity rhythm occurring at the earliest on Thursday night and at the latest on Saturday night. So although sleep debt is greater on Friday than on Thursday, circadian rhythmicity is later in “anticipation” of the weekend with the net result of later sleep timing on Friday than Thursday. BT and WT are predicted to be later on the weekend and especially so during autumn. The clock time of the circadian minimum of wake propensity is relatively stable across the week occurring ∼0.5 h later on weekends. More noticeable is that the circadian minimum occurs several hours before WT on weekends but close to WT on weekdays. This effect is more pronounced in autumn than in spring.

Panels (B) and (C) show a detailed comparison between average BT and WT measured in the field to average BT and WT and the timing of the circadian wake propensity minimum predicted by the model, for weekdays and weekends. WT for weekdays were determined by social constraints, also in the simulations. For spring, BT as predicted to within 1 h for all three BTcat during both weekdays and weekends and WT during weekends were predicted to within 1.5 h. The predicted timing of the minimum of circadian wake propensity was several hours before WT in all three BTcat during both weekdays and weekends. For autumn, sleep and wake timing were within 2 h, with the model predicting later BT and later weekend WT than measured. The circadian wake propensity minimum was located very close to WT during weekdays for all three BTcat.

## Discussion

Few ecological studies have followed light exposure, activity, and sleep patterns in young undergraduate students in their natural surroundings for continuous periods of time, i.e., three weeks, repeated over two seasons of the year (autumn and spring), including weekdays and weekends. Comparing inter-individual variability of sleep patterns across seasons and day-type by distinct reference points aims to disentangle the effects of biological, social and environmental factors in sleep regulation. Comparing differences in daily light exposure caused by DST, as well as two light exposure parameters (SN and timing of 50% LE), which are independent of each other and of DST, allowed us to test the hypothesis that human sleep follows sun time. This study can be considered a natural experiment that takes advantage of a widely institutionalized social norm (i.e., DST), and examines its association with sleep timing. Such natural experiments are considered to be of high ecological validity. Our findings provide a comprehensive assessment of individual stability and variability in these factors, and demonstrate that individual timing of sleep and activity in a modern environment with electrical lighting, largely conforms to clock time and actual light exposure (which is the combination of natural and electrical light exposure) rather than to sun time indexed by the timing of SN.

### Light Exposure

Peak light levels in the current study (around 600 in the spring and 200 lux in autumn) fall within the range of actigraphy light data (50–1000 lux) from ecological studies conducted at similar latitudes (45–51°N) (Goulet et al., 2007; Emens et al., 2009; Thorne et al., 2009; Martin et al., 2012; Van der Maren et al., 2018). Higher light levels have been reported in studies of young adults in the Rocky Mountains in Colorado (40°N), with averaged peak light levels of >2000 lux in the summer (Wright et al., 2013) and winter (Stothard et al., 2017), and extreme seasonal differences have been reported in a study of day workers in Kiruna, Sweden (68°N), with mean peak light levels during midday close to 2000 lux in summer, and around 110 lux in winter (Lowden et al., 2018). In a study of rubber tappers in the Amazon (10°39^″^ S), peak median light levels were 1200–1600 lux on work and free days, respectively (Moreno et al., 2015).

In the present study, overall 24-h light exposure was higher in spring than in autumn but did not differ between weekdays and weekends. Similar to our findings, in a study assessing weekly light exposure across seasons (winter and summer) and day-types (workdays and weekends) in young working adults in Chicago (41°88′N), the overall light exposure was lower in winter (autumn ST in our study) compared to summer (spring DST in our study), the photoperiod occurred later in the day on weekends than on weekdays, and light levels were higher in summer than in winter on weekdays and weekends during the early evening hours, likely due to the longer natural photoperiod (Crowley et al., 2015).

### Inter-Individual Variability and Stability in Sleep, Activity and Light Exposure

Several individual aspects of sleep timing were stable across seasons and weekday vs. weekends, in accordance with a previous study which besides the midpoint of sleep also analyzed stability of melatonin phase (Kantermann and Eastman, 2018).

In the current study, stability of individual differences, as assessed by intra-class correlations (ICCs), were highest for bedtime, about half the magnitude for wake time, somewhat lower for mid light exposure and minimal for overall 24-h light exposure and activity (see Tables 1, 2), indicating that within individuals, bedtime is the most consistent timing parameter. Indeed, weekday-weekend and autumn-spring bedtime correlations were highly significant, yet bedtimes and wake times were not correlated. Furthermore, categorization of individuals as early, intermediate and late based on autumn weekday bedtime predicted all timing parameters as expected; however, effects were weaker for wake time and the midpoint of activity and disappeared altogether for these timing parameters when assessed relative to the individual midpoint of light exposure. Findings suggest that bedtime in young individuals is largely tuned by individual preference (or biological determination), whereas wake time is more tuned to social constraints and environmental time cues. Similarly, in a person-centered analysis of several sleep timing and sleep continuity indicators of senior high school students, bedtime showed the highest effect size for differences between phenotype classifications (Shochat et al., 2017). In contrast, findings from a study of sleep in three preindustrial societies (Yetish et al., 2015) showed greater intra-individual variability in sleep onset than in sleep offset. These opposite findings may reflect differences in the relative contributions of social constraints and environmental time cues to sleep patterns in preindustrial vs. modern day societies (specifically here, college students). A corollary is that individual sleep duration is better predicted by individual differences in bedtime rather than wake time in the preindustrial society (Yetish et al., 2015), and is better predicted by wake time in young university students, when environmental factors and social constraints are held constant. Nevertheless, the availability of electrical evening light has been associated with later bedtime, longer sleep latency and shorter sleep duration beyond individual differences in bedtime (Wright et al., 2013; Moreno et al., 2015).

### Sleep and Activity Timing Relative to Clock Time, SN and 50% LE

Relative to **clock time**, sleep and activity timing variables were later, and sleep duration longer, on weekends compared to weekdays. Previous studies have reported later bed and wake times on weekends compared to weekends in adolescents (e.g., Tzischinsky and Shochat, 2011; Shochat et al., 2017) and young adults (e.g., Lo et al., 2014; Lowden et al., 2018). Our findings of no seasonal differences in sleep timing (during either weekdays or weekends) but significant day-type effects when measured relative to clock time are in line with other studies (Lo et al., 2014; Crowley et al., 2015). This, together with our finding of significant seasonal effects on sleep timing when measured relative to SN, confirms that social norms and constraints and associated light exposure rather than sun time determine observed sleep and activity regulation. Although in our statistical analyses we used SN as the phase marker for sun time, it is clear that using dawn and dusk would have led to a similar conclusion, i.e., sleep timing is not phase-locked to either dawn or dusk (see Figure 1). Thus, the average dusk to bedtime intervals in autumn (7-h: 56-min, and 8-h: 34-min for weekdays and weekends, respectively) are about twice those in spring (3-h: 22-min, and 3-h: 56-min), and increase from early to late bedtime categories. Similarly, dawn to wake time intervals in spring (4-h and 14-min, and 4-h and 50-min) are about twice those in autumn (1-h and 59-min, and 2-h and 36-min), and increase from early to late bedtime categories. It is evident that individual differences and social constraints override the seasonal differences in the timing of SN, dusk and dawn in determining sleep timing.

Seasonal effects on sleep timing may be evident only in higher latitudes (e.g., Friborg et al., 2014; Lowden et al., 2018). For example, in a study in Sweden (68°N) which examined sleep timing, activity, light and daytime behavioral measures in winter and summer on work days and days off, seasonal effects were found for all sleep timing variables (sleep onset, offset and mid-sleep), with later sleep timing and shorter sleep duration in winter compared to summer (Lowden et al., 2018). In another study of young adults in Norway (69 N, 39 E), sleep timing and chronotype measures were delayed in December compared to September and March (Friborg et al., 2014).

Nevertheless, season interacted with differences in bedtime category and generally showed gradually later sleep timing measures from early to intermediate to late bedtime categories. Thus, the range of bedtimes was larger in spring than in autumn, and both wake time and mid-sleep in autumn were earlier for the early category but no differences were found between the intermediate and late categories. These findings warrant further investigation of the subtle dynamics of light exposure in autumn versus spring and their effects on individual differences in sleep timing.

When expressed as “hours after” **SN**, sleep and activity timing variables were consistently later in autumn than in spring and, as for clock time, were later on weekends than on weekdays. Season interacted with day-type for wake time only, showing that later wake time in autumn than in spring was more pronounced on weekends (about 1–h and 40-m) than on weekdays (1-h only), and that weekday-weekend differences were larger in autumn (1-h and 20-min) than in spring (45-min). Similar season by bedtime category interactions were observed for the sleep timing variables as described relative to clock time.

The seasonal shift in sleep timing relative to SN (later in the spring due to DST) reflects an alignment with clock time and the significant impact of social factors rather than sun time in driving sleep timing in young college students. These findings do not support the notion that the human circadian timing system entrains to sun time as derived from an analysis of seasonal changes in self-reported sleep timing (Allebrandt et al., 2014) and the differences in sleep timing across a time zone (Roenneberg et al., 2007). Furthermore, greater weekday-weekend differences in wake time in autumn compared to spring appears to contradict findings (Kantermann et al., 2007) suggesting that sleep timing on free days as assessed by self-report, is later relative to dawn in spring (DST) than in autumn (ST) due to difficulty in tracking sun time during DST. Conversely, larger differences in sleep timing from early to late bedtime categories in spring compared to autumn in our study may lend some support to this claim. The consequences of DST shifts are still under debate (Lindenberger et al., 2018; Roenneberg et al., 2019; Skeldon and Dijk, 2019), yet our findings provide little support to the suggestion that individuals and particularly late types experience more difficulty adjusting to social constraints (clock time) in spring than in winter, due to the longer photoperiod, or to DST, or to both.

No seasonal differences were found for timing variables relative to the **midpoint of light exposure** suggesting that actual light exposure is more important than sun time. The midpoint of light exposure, but not SN, was significantly correlated with mid-sleep beyond season and day-type, and the average weekday midpoint of light exposure significantly correlated with average weekend mid-sleep (*r* = *0.46, p* = *0.049)*, suggesting that actual light exposure is a strong determinant of sleep timing (Woelders et al., 2017). Findings emphasize the importance of collecting individual light measurements, rather than measurements from satellites or ground stations, which may lead to the incorrect conclusion that light does not affect sleep timing (Porcheret et al., 2018). Weekday-weekend differences were found for wake time and mid-sleep, and season interacted with bedtime category similarly to clock time and SN reference points, with larger early to late timing intervals in spring than in autumn. Notably, although our findings show that individually determined daily light exposure, which includes both natural and electric light, is more strongly associated with sleep timing than merely sun time, they do not imply causality. Indeed, these associations may be unidirectional, bidirectional, or may involve yet a third variable, e.g., social norms and schedules. Yet even though light exposure may be driven by (rather than driving) the sleep-wake cycle, which in turn may be driven by social constraints, this light exposure will have an impact on the circadian pacemaker, as we (Skeldon et al., 2017) and others (Wright et al., 2013; Stothard et al., 2017) have shown.

### Model Simulation

The model predicts that differences in light exposure patterns, independent of other physiological differences, are sufficient to predict later sleep timing and more social jetlag for late types than intermediate types and for intermediate types than early types. This relative ordering remains, even if the imposed social constraint of wake time on weekdays is removed.

The fact that the predictions for the autumn are worse suggests that either societal factors override physiological cues or that the model is overly sensitive to the reduced day light during the autumn. Less light during the day will increase the relative importance of light during the evening, and we have not sought to take account of the high degree of individual variability in the sensitivity of the circadian system to the effects of evening light (Phillips et al., 2019).

Phase was not measured in the field, but the model predicts that the minimum of the circadian wake propensity rhythm occurs closer to wake time on weekdays than on weekends. This effect is more pronounced in the autumn than during the spring suggesting that there are seasonal differences in circadian alignment. The prediction that the minimum occurs very close to wake time during the autumn suggests wake time is during the biological night, making it particularly difficult to get up.

### Study Limitations

As in most studies, light exposure was measured from a wrist worn device and to what extent this accurately reflects retinal light exposure remains unclear. An underestimation of light exposure is particularly a concern in the autumn, due to colder temperature and long sleeves. In addition, in this study no information on the spectral composition of 24-h light exposure, which has been shown to vary across seasons (Thorne et al., 2009), was collected. As in other studies (e.g., Kantermann et al., 2007), we have used sleep timing on weekends as a proxy for circadian phase. No other accepted marker of circadian phase such as melatonin or core body temperature was recorded. This is particularly relevant because the simulation suggests major changes in the phase relationship between sleep timing and circadian rhythmicity in particular in the autumn (see Figure 6). The sample size is small, however, individual activity and light profiles over 24-h (total number of 24-h periods: 715) were collected continuously, at a 1-min rate, for two 3-week periods, in a within subject design, i.e., in each participant data were obtained in both seasons, thereby greatly increasing the statistical power. Few studies have collected such a wealth of data using wearable technology in an ecological setting. Findings may not generalize beyond college students, however, the same argument may be made for any other segment of the population. Importantly, it is likely that the daily schedules of young college students are less constrained compared to those of young adults who do not attend college and adults in later years, due to growing work and family responsibilities, and thus their sleep and activity patterns are less confounded. Indeed, population based survey findings that computed mid-sleep on free days as a proxy for chronotype, similarly to the present study, have shown that variability in chronotype reaches its peak at ages 20–24 (Fischer et al., 2017), which may reflect not only age-related changes in bio-regulatory processes of circadian phase, but also less constrained sleep schedules on free days as reported by young adults. Finally, additional seasonal differences such as temperature, which has been shown to affect sleep both in preindustrial (Yetish et al., 2015) and modern day (Hashizaki et al., 2018) societies, was not monitored here; however, in the United Kingdom, differences in temperature are minimal between autumn and spring. Thus average 24 h outdoor temperatures for autumn and spring were 8.8^∘^C and 10.8^∘^C, respectively and since most individuals spend most of their time indoors, the seasonal differences in temperature exposure are likely to have been even smaller.

## Conclusion

Our findings provide a comprehensive description of the complex interplay between environmental and individual factors that determine sleep timing in young adults. By relating sleep timing to distinct reference points, we demonstrate that beyond individual differences, sleep timing largely conforms to clock time and to actual light exposure rather than to sun time. The model simulations suggest that current models for the effects of light on the human circadian timing system perform satisfactory during spring but not so during autumn conditions and imply that assessment of circadian phase markers may provide additional insights into seasonal changes in sleep-wake regulation. Findings may inform the ongoing scientific and socio-political debate regarding the implementation of DST and its consequences on human sleep and health.

## Data Availability

The datasets generated for this study are available on request to the corresponding author.

## Ethics Statement

The research protocol received a favorable opinion from the University of Surrey Ethics Committee and was conducted in accordance with the principles of the Declaration of Helsinki. Written informed consent was obtained from all the participants prior to starting any study related procedures.

## Author Contributions

TS, NS, AS, and D-JD conceptualized the study design and protocol. NS and SF collected and processed the data. NS, PH, TS, AS, and D-JD analyzed and interpreted the data. NS, AS, and TS prepared the figures and tables. TS, NS, AS, and D-JD composed the manuscript.

## Conflict of Interest Statement

The authors declare that the research was conducted in the absence of any commercial or financial relationships that could be construed as a potential conflict of interest.

## Supplementary Material

FIGURE S1 24 h light exposure profiles of the early, intermediate and late types during weekdays and weekend in both seasons. The average 24 h light exposure profiles of the early **(A,B)**, intermediate **(C,D)**, and late **(E,F)** participants during the late autumn and late spring are shown as double plots in the figure. The horizontal bars show the average weekday (purple) and weekend (green) sleep, respectively, with the left and right error bars indicating the standard error of bed and wake times, respectively. The dashed reference lines indicate the dawn and dusk ranges during the autumn and spring. The blue and green arrows represent the average SN and midpoint of light exposure times. The horizontal-axis represents clock hour and the left vertical-axis light levels (lower panels).

FIGURE S2 24 h activity profiles of the early, intermediate and late types during weekdays and weekend in both seasons. The average 24 h activity profiles of the early **(A,B)**, intermediate **(C,D)**, and late **(E,F)** participants during the late autumn and late spring are shown as double plots in the figure. The horizontal bars show the average weekday (purple) and weekend (green) sleep, respectively, with the left and right error bars indicating the standard error of bed and wake times, respectively. The dashed reference lines indicate the dawn and dusk ranges during the autumn and spring. The blue and green arrows represent the average SN and midpoint of light exposure times. The horizontal-axis represents clock hour and the left vertical-axis activity levels (lower panels).

TABLE S1 Correlations between weekdays and weekends for mean/median BT, WT and MS times by season.

TABLE S2 Correlations between autumn and spring for mean/median BT, WT, and MS times by day-type.

TABLE S3 Correlations between mean/median BT and WT on weekdays and weekends and in autumn and spring.

TABLE S4 Averages, standard errors of the mean (SEs) of BT, WT, MS, and 50% act by season, day type, and BT category (early, intermediate, late) relative to clock time.

TABLE S5 Averages, standard errors of the mean (SEs) of BT, WT, MS and 50% act, by season, day type, and BT category (early, intermediate, late) relative to solar noon (SN).

TABLE S6 Averages, standard errors of the mean (SEs) of BT, WT, MS and 50% act, by season, day type, and BT category (early, intermediate, late) after 50% light exposure (LE).

## References

[B1] Allebrandt K. V. Teder-Laving M. Kantermann T. Peters A. Campbell H. Rudan I. (2014). Chronotype and sleep duration: the influence of season of assessment. *Chronobiol. Int.* 31 731–740. 10.3109/07420528.2014.901347 24679223

[B2] Burgess H. J. Molina T. A. (2014). Home lighting before usual bedtime impacts circadian timing: a field study. *Photochem. Photobiol.* 90 723–726. 10.1111/php.12241 24918238 PMC4053688

[B3] Chinoy E. D. Duffy J. F. Czeisler C. A. (2018). Unrestricted evening use of light-emitting tablet computers delays self-selected bedtime and disrupts circadian timing and alertness. *Physiol. Rep.* 6:e13692. 10.14814/phy2.13692 29845764 PMC5974725

[B4] Crowley S. J. Molina T. A. Burgess H. J. (2015). A week in the life of full-time office workers: work day and weekend light exposure in summer and winter. *Appl. Ergon.* 46 193–200. 10.1016/j.apergo.2014.08.006 25172304 PMC4185224

[B5] Crowley S. J. Wolfson A. R. Tarokh L. Carskadon M. A. (2018). An update on adolescent sleep: new evidence informing the perfect storm model. *J. Adolesc.* 67 55–65. 10.1016/j.adolescence.2018.06.001 29908393 PMC6054480

[B6] Daan S. Merrow M. Roenneberg T. (2002). External time-internal time. *J. Biol. Rhythms* 17 107–109. 10.1177/074873002129002375 12002157

[B7] Dijk D. Czeisler C. (1995). Contribution of the circadian pacemaker and the sleep homeostat to sleep propensity, sleep structure, electroencephalographic slow waves, and sleep spindle activity in humans. *J. Neurosci.* 15 3526–3538. 10.1523/JNEUROSCI.15-05-03526.1995 7751928 PMC6578184

[B8] Duffy J. F. Cain S. W. Chang A.-M. Phillips A. J. K. Munch M. Y. Gronfier C. (2011). Sex difference in the near-24-hour intrinsic period of the human circadian timing system. *Proc. Natl. Acad. Sci. U.S.A.* 108 15602–15608. 10.1073/pnas.1010666108 21536890 PMC3176605

[B9] Duffy J. F. Wright K. P. (2005). Entrainment of the human circadian system by light. *J. Biol. Rhythms* 20 326–338. 10.1177/0748730405277983 16077152

[B10] Emens J. S. Yuhas K. Rough J. Kochar N. Peters D. Lewy A. J. (2009). Phase angle of entrainment in morning and evening-types under naturalistic conditions. *Chronobiol. Int.* 26 474–493. 10.1080/07420520902821077 19360491 PMC2699216

[B11] Fischer D. Lombardi D. A. Marucci-Wellman H. Roenneberg T. (2017). Chronotypes in the US-Influence of age and sex. *PLoS One* 12:e0178782. 10.1371/journal.pone.0178782 28636610 PMC5479630

[B12] Friborg O. Bjorvaten B. Amponsah B. Pallesen S. (2012). Associations between seasonal variations in day length (photoperiod), sleep timing, sleep quality and mood: a comparison between Ghana (5^∘^) and Norway (69^∘^). *J. Sleep Res.* 21 176–184. 10.1111/j.1365-2869.2011.00982.x 22074234

[B13] Friborg O. Rosenvinge J. H. Wynn R. Gradisar M. (2014). Sleep timing, chronotype, mood, and behavior at an arctic latitude (69^∘^N). *Sleep Med.* 15 798–807. 10.1016/j.sleep.2014.03.014 24933084

[B14] Goulet G. Mongrain V. Desrosiers C. Paquet J. Dumont M. (2007). Daily light exposure in morning-type and evening-type individuals. *J. Biol. Rhythms* 22 151–158. 10.1177/0748730406297780 17440216

[B15] Gradisar M. Crowley S. J. (2013). Delayed sleep phase disorder in youth. *Curr. Opin. Psychiatry* 26 580–585. 10.1097/YCO.0b013e328365a1d4 24060912 PMC4142652

[B16] Hagenauer M. H. Perryman J. I. Lee T. M. Carskadon M. A. (2009). Adolescent changes in the homeostatic and circadian regulation of sleep. *Dev. Neurosci.* 31 276–284. 10.1159/000216538 19546564 PMC2820578

[B17] Hashizaki M. Nakajima H. Shiga T. Tsutsumi M. Kume K. (2018). A longitudinal large-scale objective sleep data analysis revealed a seasonal sleep variation in the Japanese population. *Chronobiol. Int.* 35 933–945. 10.1080/07420528.2018.1443118 29589960

[B18] Kantermann T. Burgess H. J. (2017). Average mid-sleep time as a proxy for circadian phase. *PsyCh J.* 6 290–291. 10.1002/pchj.182 29035008

[B19] Kantermann T. Eastman C. I. (2018). Circadian phase, circadian period and chronotype are reproducible over months. *Chronobiol. Int.* 35 280–288. 10.1080/07420528.2017.1400979 29148844 PMC6055478

[B20] Kantermann T. Juda M. Merrow M. Roenneberg T. (2007). The human circadian clock’s seasonal adjustment is disrupted by daylight saving time. *Curr. Biol.* 17 1996–2000. 10.1016/j.cub.2007.10.025 17964164

[B21] Lindenberger L. M. Ackermann H. Parzeller M. (2018). The controversial debate about daylight saving time (DST)—results of a retrospective forensic autopsy study in Frankfurt/Main (Germany) over 10 years (2006–2015). *Int. J. Legal Med.* 133 1259–1265. 10.1007/s00414-018-1960-z 30386873

[B22] Lo J. C. Leong R. L. F. Loh K. K. Dijk D. J. Chee M. W. L. (2014). Young adults’ sleep duration on work days: differences between East and West. *Front. Neurol.* 5:81. 10.3389/fneur.2014.00081 24904524 PMC4036075

[B23] Lowden A. Lemos N. Gonçalves B. Öztürk G. Louzada F. Pedrazzoli M. (2018). Delayed sleep in winter related to natural daylight exposure among arctic day workers. *Clocks Sleep* 1 105–116. 10.3390/clockssleep1010010 PMC7509675 33089157

[B24] Martin J. S. Hébert M. Ledoux E. Gaudreault M. Laberge L. (2012). Relationship of chronotype to sleep, Light exposure, and work-related fatigue in student workers. *Chronobiol. Int.* 29 295–304. 10.3109/07420528.2011.653656 22390242

[B25] Moreno C. R. C. Vasconcelos S. Marqueze E. C. Lowden A. Middleton B. Fischer F. M. (2015). Sleep patterns in Amazon rubber tappers with and without electric light at home. *Sci. Rep.* 5:14074. 10.1038/srep14074 26361226 PMC4566125

[B26] Phillips A. J. K. Vidafar P. Burns A. C. McGlashan E. M. Anderson C. Rajaratnam S. M. W. (2019). High sensitivity and interindividual variability in the response of the human circadian system to evening light. *Proc. Natl. Acad. Sci. U.S.A.* 116 12019–12024. 10.1073/pnas.1901824116 31138694 PMC6575863

[B27] Phillips A. J. K. K. Chen P. Y. Robinson P. A. (2010). Probing the mechanisms of chronotype using quantitative modeling. *J. Biol. Rhythms* 25 217–227. 10.1177/0748730410369208 20484693

[B28] Porcheret K. Wald L. Fritschi L. Gerkema M. Gordijn M. Merrrow M. (2018). Chronotype and environmental light exposure in a student population. *Chronobiol. Int.* 35 1365–1374. 10.1080/07420528.2018.1482556 29913073 PMC6234547

[B29] Roenneberg T. (2013). Chronobiology: the human sleep project. *Nature* 498 427–428. 10.1038/498427a 23803826

[B30] Roenneberg T. Kuehnle T. Pramstaller P. P. Ricken J. Havel M. Guth A. (2004). A marker for the end of adolescence. *Curr. Biol.* 14 R1038–R1039. 10.1016/j.cub.2004.11.039 15620633

[B31] Roenneberg T. Kumar C. J. Merrow M. (2007). The human circadian clock entrains to sun time. *Curr. Biol.* 17 R44–R45. 10.1016/j.cub.2006.12.011 17240323

[B32] Roenneberg T. Wirz-Justice A. Skene D. J. Ancoli-Israel S. Wright K. P. Dijk D.-J. (2019). Why should we abolish daylight saving time? *J. Biol. Rhythms* 34 227–230. 10.1177/0748730419854197 31170882 PMC7205184

[B33] Shochat T. Barker D. H. Sharkey K. M. Van Reen E. Roane B. M. Carskadon M. A. (2017). An approach to understanding sleep and depressed mood in adolescents: person-centred sleep classification. *J. Sleep Res.* 26 709–717. 10.1111/jsr.12550 28573658 PMC5705436

[B34] Skeldon A. C. Derks G. Dijk D.-J. J. (2016). Modelling changes in sleep timing and duration across the lifespan: changes in circadian rhythmicity or sleep homeostasis? *Sleep Med. Rev.* 28 96–107. 10.1016/j.smrv.2015.05.011 26545247

[B35] Skeldon A. C. Dijk D.-J. (2019). School start times and daylight saving time confuse California lawmakers. *Curr. Biol.* 29 R278–R279. 10.1016/j.cub.2019.03.014 31014483

[B36] Skeldon A. C. Phillips A. J. K. Dijk D.-J. (2017). The effects of self-selected light-dark cycles and social constraints on human sleep and circadian timing: a modeling approach. *Sci. Rep.* 7:45158. 10.1038/srep45158 28345624 PMC5366875

[B37] Stothard E. R. McHill A. W. Depner C. M. Birks B. R. Moehlman T. M. Ritchie H. K. (2017). Circadian entrainment to the natural light-dark cycle across seasons and the weekend. *Curr. Biol.* 27 508–513. 10.1016/j.cub.2016.12.041 28162893 PMC5335920

[B38] Thorne H. C. Jones K. H. Peters S. P. Archer S. N. Dijk D. J. (2009). Daily and seasonal variation in the spectral composition of light exposure in humans. *Chronobiol. Int.* 26 854–866. 10.1080/07420520903044315 19637047

[B39] Tzischinsky O. Shochat T. (2011). Eveningness, sleep patterns, daytime functioning, and quality of life in israeli adolescents. *Chronobiol. Int.* 28 338–343. 10.3109/07420528.2011.560698 21539425

[B40] Van der Maren S. Moderie C. Duclos C. Paquet J. Daneault V. Dumont M. (2018). Daily profiles of light exposure and evening use of light-emitting devices in young adults complaining of a delayed sleep schedule. *J. Biol. Rhythms* 33 192–202. 10.1177/0748730418757007 29463186

[B41] Wilson J. Reid K. J. Braun R. I. Abbott S. M. Zee P. C. (2018). Habitual light exposure relative to circadian timing in delayed sleep-wake phase disorder. *Sleep* 41:zsy166. 10.1093/sleep/zsy166/5078613 30423177 PMC6231529

[B42] Wittmann M. Dinich J. Merrow M. Roenneberg T. (2006). Social Jetlag: misalignment of biological and social time. *Chronobiol. Int.* 23 497–509. 10.1080/07420520500545979 16687322

[B43] Woelders T. Beersma D. G. M. Gordijn M. C. M. Hut R. A. Wams E. J. (2017). Daily light exposure patterns reveal phase and period of the human circadian clock. *J. Biol. Rhythms* 32 274–286. 10.1177/0748730417696787 28452285 PMC5476188

[B44] Wright K. P. McHill A. W. Birks B. R. Griffin B. R. Rusterholz T. Chinoy E. D. (2013). Entrainment of the human circadian clock to the natural light-dark cycle. *Curr. Biol.* 23 1554–1558. 10.1016/j.cub.2013.06.039 23910656 PMC4020279

[B45] Yetish G. Kaplan H. Gurven M. Wood B. Pontzer H. Manger P. R. (2015). Natural sleep and its seasonal variations in three pre-industrial societies. *Curr. Biol.* 25 2862–2868. 10.1016/j.cub.2015.09.046 26480842 PMC4720388

[B46] Zeitzer J. M. Dijk D.-J. Kronauer R. E. Brown E. N. Czeisler C. A. (2000). Sensitivity of the human circadian pacemaker to nocturnal light: melatonin phase resetting and suppression. *J. Physiol.* 526 695–702. 10.1111/j.1469-7793.2000.00695.x 10922269 PMC2270041

