# Supplementary figures and images for: Sleep Timing in Late Autumn and Late Spring Associates With Light Exposure Rather Than Sun Time in College Students
Source: Front Neurosci. 2019 Aug 28;13:882. doi: 10.3389/fnins.2019.00882 (PMC6724614; doi:10.3389/fnins.2019.00882)

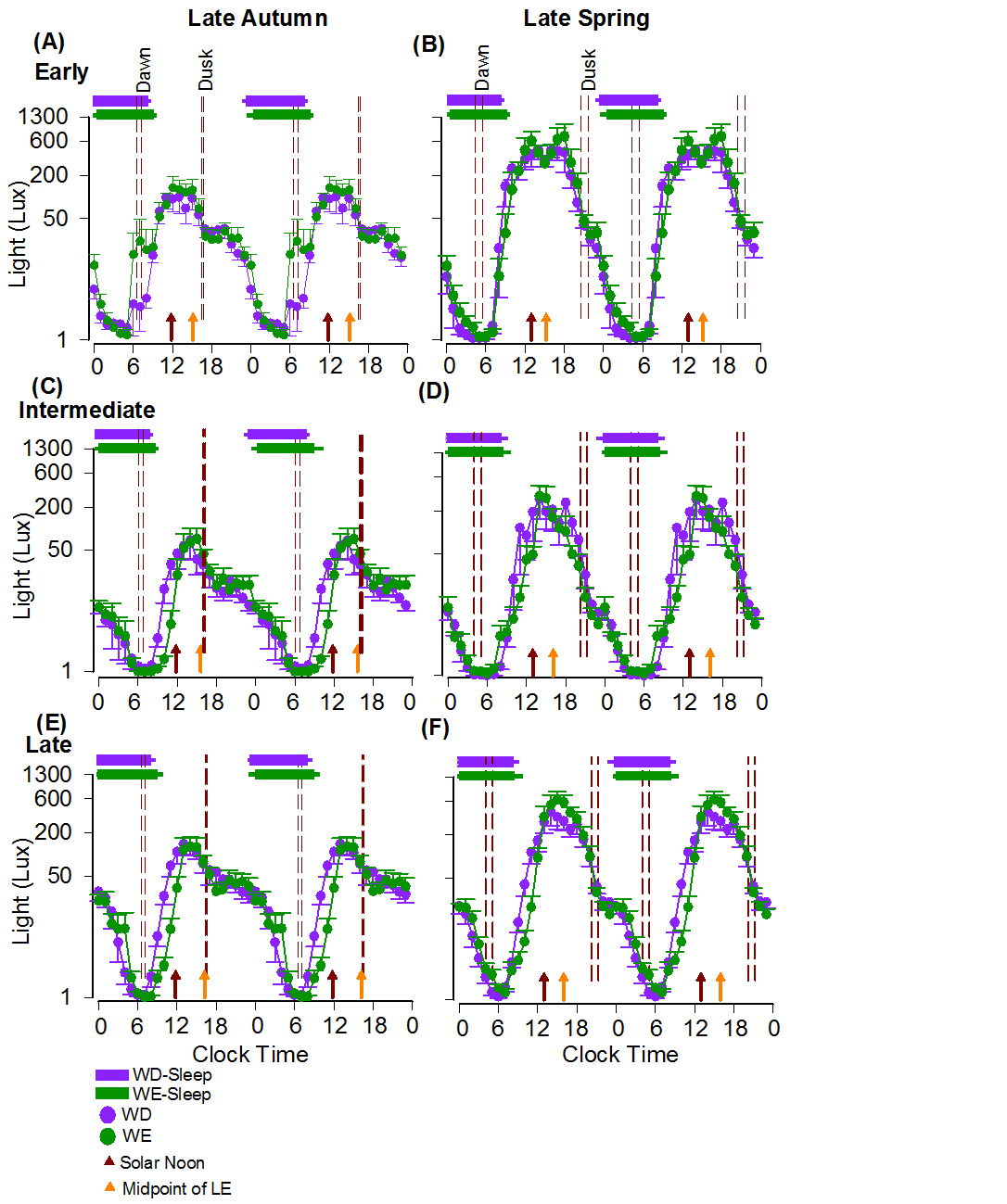

Supplement: FIGURE S1 — 24 h light exposure profiles of the early, intermediate and late types during weekdays and weekend in both seasons. The average 24 h light exposure profiles of the early (A,B), intermediate (C,D), and late (E,F) participants during the late autumn and late spring are shown as double plots in the figure. The horizontal bars show the average weekday (purple) and weekend (green) sleep, respectively, with the left and right error bars indicating the standard error of bed and wake times, respectively. The dashed reference lines indicate the dawn and dusk ranges during the autumn and spring. The blue and green arrows represent the average SN and midpoint of light exposure times. The horizontal-axis represents clock hour and the left vertical-axis light levels (lower panels). [file Image_1.JPEG]

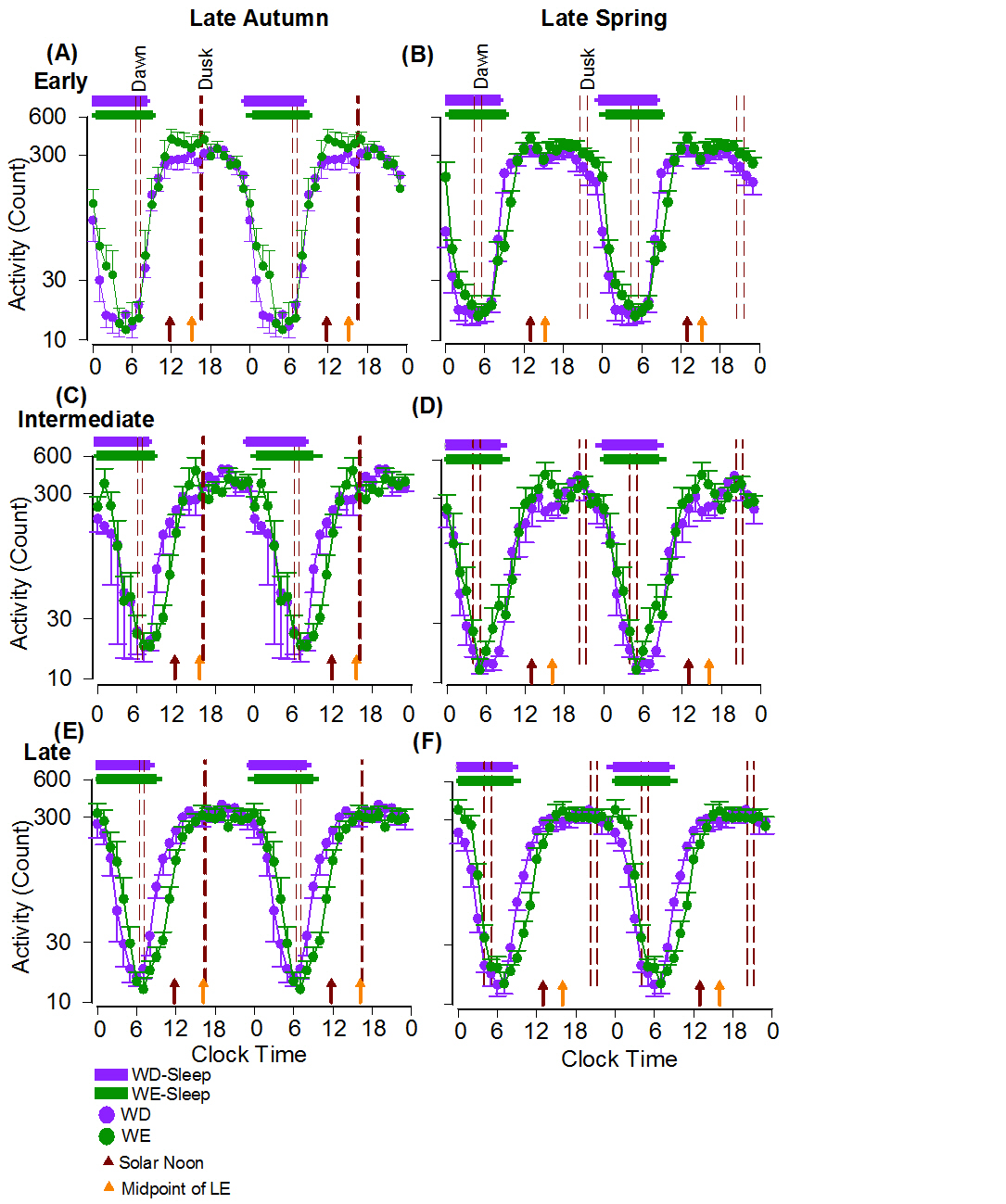

Supplement: FIGURE S2 — 24 h activity profiles of the early, intermediate and late types during weekdays and weekend in both seasons. The average 24 h activity profiles of the early (A,B), intermediate (C,D), and late (E,F) participants during the late autumn and late spring are shown as double plots in the figure. The horizontal bars show the average weekday (purple) and weekend (green) sleep, respectively, with the left and right error bars indicating the standard error of bed and wake times, respectively. The dashed reference lines indicate the dawn and dusk ranges during the autumn and spring. The blue and green arrows represent the average SN and midpoint of light exposure times. The horizontal-axis represents clock hour and the left vertical-axis activity levels (lower panels). [file Image_2.JPEG]
