# Supplementary material for: Sleep Timing in Late Autumn and Late Spring Associates With Light Exposure Rather Than Sun Time in College Students
Source: Front Neurosci. 2019 Aug 28;13:882. doi: 10.3389/fnins.2019.00882 (PMC6724614; doi:10.3389/fnins.2019.00882)
Supplement: TABLE S1 — Correlations between weekdays and weekends for mean/median BT, WT and MS times by season. [file Table_1.DOCX]

**Supplemental Tables:**

**S-table 1:** Correlations between weekdays and weekends for mean/median BT, WT and MS times by season

| Season | Weekday-Weekend BT-mean/median | Weekday-Weekend WT-mean/median | Weekday-Weekend MS-mean/median |
| --- | --- | --- | --- |
| Spring | 0.95***/0.94*** | 0.75**/0.78*** | 0.88***/0.88*** |
| Autumn | 0.91***/0.88*** | 0.77***/0.54* | 0.71**/0.74** |

***p<.0001 **p<.001 *p<.05. Means and medians of BT, WT and MS were computed per participant by season and day-type; then correlated between weekday and weekend

**S-Table 2:** Correlations between autumn and spring for mean/median BT, WT, and MS times by day-type

| Daytype | Autumn-Spring BT-mean/median | Autumn-Spring WT-mean/median | Autumn-Spring MS-mean/median |
| --- | --- | --- | --- |
| Weekday | 0.88***/0.89*** | 0.61**/0.54* | 0.81***/0.83*** |
| Weekend | 0.72**/0.76** | 0.85***/0.76** | 0.84***/0.82*** |

 ***p<.0001 **p<.001 *p<.05. Means and medians of BT, WT and MS were computed per participant by season and day-type; then correlated between autumn and spring.

**S- Table -3:** Correlations between mean/median BT and WT on weekdays and weekends and in autumn and spring

| BT-WT Weekday Mean/median | BT-WT Weekend Mean/median | BT-WT Autumn  Mean/median | BT-WT Spring  Mean/median |
| --- | --- | --- | --- |
| 0.32/ 0.42^ | 0.27/ 0.23 | 0.33/ 0.39^ | 0.31/0.42^ |

^p<.1. Bed- and wake times were correlated per participant by season and day type, and both mean and medians of the correlations were computed across participants.

**S-Table 4:** Averages, standard errors of the mean (SEs) of BT, WT, MS, and 50% act by season, day type, and BT category (early, intermediate, late) relative to **CLOCKTIME.**

|  | Autumn BT (hh:mm) | Spring BT (hh:mm) | Autumn WT (hh:mm) | Spring WT (hh:mm) | Autumn MS (hh:mm) | Spring MS (hh:mm) | Autumn 50%act (hh:mm) | Spring 50%act (hh:mm) | BT | WT | MS | 50% act |
| --- | --- | --- | --- | --- | --- | --- | --- | --- | --- | --- | --- | --- |
| Early | 23:58± 0:13  WD: 23:33±0:13  WE: 24:26±0:16** | 23:17 ± 0:13*  WD:  23:02 ±0:14*  WE:  23:30± 0:17* | 08:49±0:14  WD:  08:22±0:14  WE:  09:14±0:17** | 08:25±0:14*  WD: 08:08±0:15  WE: 08:45±0:17 ** | 04:33±0:10  WD:  04:10±0:11  WE:  04:55±0:13 ** | 04:13±0:10*  WD:  03:59±0:11  WE:  04:28±0:14 ** | 16:17±0:23  WD:  15:49±0:25  WE:  16:42±0:33 | 15:39±0:16  WD:  15:10±0:17  WE:  16:10±0:21** | SEASON:  F(1,64)=0.02, p=0.8860  **DAY-TYPE:**  **F(1,64)=19.26, p<0.0001**  **BTcat:^**  **F(2,64)=38.95, p<0.0001**  **SEASON*BTcat:**  **F(2,64)=9.71, p=0.0002**  No interactions with DAY-TYPE | SEASON:  F(1,64)=2.65, p=0.1087  **DAY-TYPE:**  **F(1,64)=63.67, p<0.0001**  **BTcat:^^**  **F(2,64)=11.68, p<0.0001**  **SEASON*BTcat:**  **F(2,64)=3.49, p=0.0363**  **SEASON*DAY-TYPE: F(2,64)=6.12, p=0.0161**  **DAY-TYPE*BTcat:**  **F(2,64)=3.85, p=0.0264** | SEASON:  F(1,62)=0.07, p=0.7862  **DAY-TYPE:**  **F(1,62)=55.45, p<0.0001**  **BTcat:^**  **F(2,62)=43.32, p<0.0001**  **SEASON*BTcat*:**  **F(2,62)=3.93, p=0.0248**  No interactions with DAY-TYPE. | SEASON:  F(1,48)=1.85, p=0.1796  **DAY-TYPE:**  **F(1,48)=6.91, p=0.0115**  **BTcat:^^**  **F(2,48)=6.80, p=0.0025**  No interactions with SEASON  **DAYTYPE*BTcat:**  **F(2,48)=5.17, p=0.0093** |
| Intermediate | 24:49±0:17  WD: 24:36±0:19  WE:  24:58± 0:23 | 01:11±0:17  WD: 24:55±0:19  WE:  01:34±0:23 | 09:46±0:19  WD:  08:56±0:20  WE:  10:46±0:23** | 09:17±0:19  WD: 09:11±0:20  WE: 09:14±0:23* | 05:46±0:14  WD:  05:09±0:15  WE:  06:24±0:18 ** | 05:40±0:14  WD:  05:21±0:15  WE:  05:55±0:18 | 16:51±0:23  WD:  16:55±0:23  WE:  17:02±0:32 | 16:58±0:22  WD:  17:16±0:23  WE:  16:29±0:29 |  |  |  |  |
| Late | 01:53± 0:13  WD:  01:37 ±0:14  WE:  02:10±0:17**, ^ | 02:15±0:13  WD: 02:03±0:14  WE:  02:28± 0:17  ^ | 09:56±0:14  WD:  09:03±0:16  WE:  10:46±0:18**, ^^ | 10:10±0:14  WD: 09:31±0:16  WE: 10:51±0:18  **, ^ | 06:08±0:10  WD:  05:37±0:11  WE:  06:38±0:13  **, ^^ | 06:28:11  WD:  06:02±0:12  WE:  06:55±0:14 **, ^ | 17:31±0:20  WD:  17:09±0:21  WE:  17:44±0:29 | 17:13±0:16  WD:  16:45±0:17  WE:  17:44±0:22** |  |  |  |  |

*Differences between autumn and spring. **Differences between Weekday (WD) and Weekend (WE).

^ Early<Intermediate<Late. ^^ Early<Intermediate=Late.

**S-Table 5:** Averages, standard errors of the mean (SEs) of BT, WT, MS and 50%act, by season, day type, and BT category (early, intermediate, late) relative to **SOLAR NOON (SN).**

|  | Autumn BT after SN (hrs) | Spring BT after SN (hrs) | Autumn WT after SN (hrs) | Spring WT after SN (hrs) | Autumn MS after SN (hrs) | Spring MS after SN (hrs) | Autumn 50%act after SN (hrs) | Spring 50%act after SN (hrs) | BT after SN | WT after SN | MS after SN | 50% act after SN |
| --- | --- | --- | --- | --- | --- | --- | --- | --- | --- | --- | --- | --- |
| Early | 12.14±0.21  WD: 11.72±0.23  WE: 12.61±0.27** | 10.32±0.22*  WD:  10.06 ±0.23*  WE: 10.53±0.28* | 20.99±0.22  WD:  20.53±0.24  WE:  21.40±0.28** | 19.44±023*  WD:  19.15±0.25*  WE:  19.77±0.29*^,^  ** | 16.61±0.15  WD:  16.53±0.16  WE:  16.70±0.21 | 15.24±0.15*  WD:  15.03±0.16*  WE:  15.46±0.21* | 4.43±0.39  WD:  3.96±0.41  WE:  4.84±0.56 | 2.64±0.28*  WD:  2.10±0.29  WE:  3.21±0.36 | **SEASON:**  **F(1,64)=79.87, p<0.0001**  **DAYTYPE:**  **F(1,64)=19.05, p<0.0001**  **BTcat: ^**  **F(2,64)=38.80, p<0.0001**  **SEASON*BTcat:**  **F(2,64)=9.42, p=0.0003**  No interactions with day-type. | **SEASON:**  **F(1,64)=106.09, p<0.0001**  **DAYTYPE:**  **F(1,64)=63.21, p<0.0001**  **BTcat: ^^**  **F(2,64)=11.73, p<0.0001**  **SEASON*BTcat:**  **F(2,64)=3.52, p=0.0354**  **DAYTYPE*BTcat: F(1,64)=3.81, p=0.0273**  **SEASON*DAYTYPE: F(1,64)=5.97, p=0.0173** | **SEASON:**  **F(1,62)=109.01, p<0.0001**  **DAYTYPE:**  **F(1,62)=10.49, p=0.0019**  **BTcat:^**  **F(2,62)=54.94, p<0.0001**  **SEASON*BTcat:**  **F(2,62)=6.15, p=0.0037**  No interactions with day-type. | **SEASON:**  **F(1,48)=51.00, p<0.0001**  **DAYTYPE:**  **F(1,48)=7.36, p=0.0092**  **BTcat: ^^**  **F(2,48)=6.71, p<0.0027**  No interactions with season.  **DAYTYPE*Btcat:**  **F(2,48)=5.44, p=0.0074** |
| Intermediate | 13.00±0.30  WD:  12.78±0.32  WE:  13.15±0.38 | 12.21±0.29*  WD: 11.92±0.31*  WE: 12.57±0.38 | 21.96±0.32  WD:  21.13±0.34  WE:  22.94±0.39** | 20.30±0.32*  WD:  20.21±0.34*  WE:  20.26±0.39* | 17.63±0.23  WD:  17.44±0.25  WE:  17.87±0.33 | 16.27±0.23*  WD:  16.06±0.25*  WE:  16.43±0.31* | 4.99±0.38  WD:  5.04±0.39  WE:  5.23±0.54 | 3.83±0.37*  WD:  4.17±0.39  WE:  3.31±0.49 |  |  |  |  |
| Late | 14.07±0.22  WD:  13.79±0.24  WE:  14.34±0.29  ^ | 13.28±0.22*  WD: 13.07±0.24*  WE: 13.48±0.29*  ^ | 22.10±0.24  WD:  21.24±0.26  WE:  22.93±0.30**, ^^ | 21.19±0.24*  WD:  20.54±0.25*  WE:  21.87±0.30*^,^  **, ^ | 18.13±0.14  WD:  18.03±0.15  WE:  18.21±0.20  ^^ | 17.44±0.15*  WD:  17.17±0.16*  WE:  17.73±0.20**, ^ | 5.69±0.34  WD:  5.33±0.35  WE:  5.90±0.49 | 4.22±0.28*  WD:  3.75±0.29  WE:  4.75±0.36 |  |  |  |  |

*Differences between autumn and spring. **Differences between Weekday (WD) and Weekend (WE).

^ Early<Intermediate<Late. ^^ Early<Intermediate=Late.

**S-Table 6:** Averages, standard errors of the mean (SEs) of BT, WT, MS and 50% act, by season, day type, and BT category (early, intermediate, late) after **50% light exposure (LE).**

|  | Autumn BT after 50% LE (hrs) | Spring BT after 50% LE (hrs) | Autumn WT after 50% LE (hrs) | Spring WT after 50% LE (hrs) | Autumn MS after 50% LE (hrs) | Spring MS after 50% LE (hrs) | Autumn 50%act after 50% LE (hrs) | Spring 50%act after 50% LE (hrs) | BT after 50% LE | WT after 50% LE | MS after 50% LE | 50% act after 50% LE |
| --- | --- | --- | --- | --- | --- | --- | --- | --- | --- | --- | --- | --- |
| Early | 8.68±0.26  WD:  8.48±0.27  WE:  8.98±0.35 | 8.04± 0.26*  WD:  7.93±0.27*  WE:  8.04±0.35* | 17.56±0.30  WD:  17.27±0.33  WE:  17.84±0.40 | 17.20±0.31  WD:  17.08±0.34  WE:  17.33±0.41 | 13.38± 0.23  WD:  13.25±0.24  WE:  13.53±0.34 | 12.94±0.23  WD:  12.87±0.24  WE:  12.97±0.34 | 1.01±0.35  WD:  0.94±0.38  WE:  0.98±0.53 | 0.43±0.23  WD:  0.01±0.25*  WE:  0.89±0.33** | SEASON:  F(1,64)=0.21, p=0.6497  DAY-TYPE:  F(1,64)=1.05, p=0.3089  **BTcat:^^**  **F(2,64)=11.35, p<0.0001**  **SEASON*BTcat: F(2,64)=6.24, p=0.0033**  No interactions with DAY-TYPE | SEASON:  F(1,64)=0.97, p=0.3280  **DAY-TYPE:**  **F(1,64)=9.71, P=0.0027**  BTcat:  F(2,64)=0.48, P=0.6187  **SEASON*BTcat: F(2,64)=5.38, p=0.0069**  No interactions with DAY-TYPE | SEASON:  F(1,62)=0.38, p=0.5405  **DAY-TYPE:**  **F(1,62)=6.16, p=0.0158**  **BTcat: ^^ F(2,62)=5.37, p=0.0070**  **SEASON*BTcat: F(2,62)=6.13, p=0.0037**    No interactions with DAY-TYPE | SEASON:  F(1,48)=2.25, p=0.1399  DAY-TYPE:  F(1,48)=0.00, p=0.9764  BTcat: F(2,48)=1.10, p=0.3427  No interactions with season  **DAY-TYPE* BTcat:**  **F(2,48)=3.39, p=0.0420** |
| Intermediate | 9.19±0.37  WD:  9.13±0.37  WE:  8.96±0.53 | 9.14± 0.35  WD:  8.79.37  WE:  9.66±0.48 | 17.99±0.43  WD:  17.47±0.45  WE:  18.70±0.61*^,^** | 17.18±0.42*  WD:  17.08±0.45  WE:  17.16±0.55 | 14.06± 0.32  WD:  13.71±0.32  WE:  14.36±0.50 | 13.58±0.31  WD:  13.22±0.32  WE:  13.98±0.46 | 1.08±0.32  WD:  1.43±0.32  WE:  0.93±0.50 | 0.88±0.31  WD:  1.16±0.33  WE:  0.47±0.44 |  |  |  |  |
| Late | 9.72±0.27  WD:  9.84±0.28  WE:  9.59±0.38  ^^^^ | 10.20±0.27*  WD:  10.28±0.28  WE:  10.12±0.36  ^^^ | 17.47±0.33  WD:  17.03±0.35  WE:  17.83±0.44 | 18.07±0.32*  WD:  17.72±0.34*  WE:  18.47±0.41 | 13.76±0.24  WD:  13.56±0.25  WE:  13.94±0.36 | 14.37±0.24*  WD:  14.19±0.26*  WE:  14.55±0.35  ^^^ | 1.25±0.30  WD:  1.36±0.31  WE:  1.06±0.46 | 1.15±0.23  WD:  0.94±0.25  WE:  1.38±0.33 |  |  |  |  |

*Differences between autumn and spring. **Differences between Weekday (WD) and Weekend (WE).

^ Early<Intermediate<Late. ^^ Early<Intermediate=Late. ^^^Early=Intermediate<Late. ^^^^Early<Late, Early=Intermediate, Late=Intermediate.
